# High-Sensitivity Piezoelectric MEMS Accelerometer for Vector Hydrophones

**DOI:** 10.3390/mi14081598

**Published:** 2023-08-14

**Authors:** Shuzheng Shi, Liyong Ma, Kai Kang, Jie Zhu, Jinjiang Hu, Hong Ma, Yongjun Pang, Zhanying Wang

**Affiliations:** 1 School of Mechanical Engineering, Hebei University of Architecture, Zhangjiakou 075000, China; shishuzheng2000@163.com (S.S.); maliyong@buaa.edu.cn (L.M.); kangkai_jgxy@163.com (K.K.); 15933136690@163.com (J.H.); mahong0210@163.com (H.M.); 2 HBIS Group Co., Ltd., Shijiazhuang 050023, China; 3 School of Computer Science and Engineering, North China Institute of Aerospace Engineering, Langfang 065000, China; zhujie0424@126.com; 4 School of Materials Science and Engineering, University of Science and Technology Beijing, Beijing 100083, China

**Keywords:** microelectromechanical systems, cantilever beam, piezoelectric accelerometer, vector hydrophone, sensitivity

## Abstract

In response to the growing demand for high-sensitivity accelerometers in vector hydrophones, a piezoelectric MEMS accelerometer (PMA) was proposed, which has a four-cantilever beam integrated inertial mass unit structure, with the advantages of being lightweight and highly sensitive. A theoretical energy harvesting model was established for the piezoelectric cantilever beam, and the geometric dimensions and structure of the microdevice were optimized to meet the vibration pickup conditions. The sol-gel and annealing technology was employed to prepare high-quality PZT thin films on silicon substrate, and accelerometer microdevices were manufactured by using MEMS technology. Furthermore, the MEMS accelerometer was packaged for testing on a vibration measuring platform. Test results show that the PMA has a resonant frequency of 2300 Hz. In addition, there is a good linear relationship between the input acceleration and the output voltage, with *V* = 8.412*a* − 0.212. The PMA not only has high sensitivity, but also has outstanding anti-interference ability. The accelerometer structure was integrated into a vector hydrophone for testing in a calibration system. The results show that the piezoelectric vector hydrophone (PVH) has a sensitivity of –178.99 dB@1000 Hz (0 dB = 1 V/μPa) and a bandwidth of 20~1100 Hz. Meanwhile, it exhibits a good “8” shape directivity and consistency of each channel. These results demonstrate that the piezoelectric MEMS accelerometer has excellent capabilities suitable for use in vector hydrophones.

## 1. Introduction

Accurately extracting information from underwater acoustic sources is a crucial aspect of sonar system research. Hydrophones serve as a window for communication between sonar systems and the ocean, acting as the “eyes and ears” of the sonar system. A hydrophone is a transducer to convert the acoustic radiation signals of underwater targets into electrical signals, which is commonly used for transmitting and receiving acoustic signals [1,2,3]. A vector hydrophone can measure vector information, such as the direction of underwater targets, particle velocity, displacement, and acceleration of water particles, while maintaining spatial colocation and time synchronization [4,5]. Due to its excellent sensitivity and directional capabilities, the vector hydrophone is widely used for detecting low-frequency underwater target signals. According to the measurement principle, the vector hydrophone can be divided into two types: pressure difference type [6] and resonance type [7]. Compared to the pressure difference type, the resonance type sensor can directly measure the movement of the sound wave and has advantages such as reliable performance, high sensitivity, and good low-frequency directivity. A better vibration design scheme is to install a sensor along the orthogonal coordinate axis inside a rigid shell to pick up the vibration signal of the water particle. The accelerometer is suitable for a vector hydrophone, because its output characteristic is dipole, and it is possible to measure low frequencies. It requires that the vibration pickup should have a small enough mass to approach neutral buoyancy and resonate with the water medium to pick up underwater vibration signals. In 2023, Mireles, J. et al. [8] developed a MOEMS accelerometer using SOI technology, which contains a mass structure and handle layers coupled with four designed springs built. The sensor exhibited a resonant frequency of 1274 Hz under running conditions up to 7 g. However, the sensitivity of the low-frequency response is lower, as the sensitivity is reduced by 6 dB for each doubling of the frequency. Therefore, a resonance type vector hydrophone requires a lightweight, compact, and highly sensitive micro accelerometer as the vibration pickup unit [9]. The MEMS accelerometer is smaller than a traditional accelerometer, reducing the impact of the original acoustic field when used in the manufacture of hydrophones, and making the detection more accurate. Meanwhile, it has lighter weight, lower manufacturing cost, and easier-to-achieve low-frequency target detection [10].

Currently, there are three types of MEMS accelerometers: piezoresistive [11], capacitive [12], and piezoelectric [13]. The piezoresistive MEMS accelerometer is an early developed type of miniaturized accelerometer. It consists of a cantilever beam with a piezoresistive material and an inertial mass unit, which possesses the advantages of simple structure and relatively easy fabrication. However, the large temperature coefficient of the piezoresistive material results in the sensitivity of the acceleration detection performance to temperature and the poor anti-interference ability. Additionally, it has low sensitivity, significant hysteresis effects, and a high-power dependence, making it difficult to apply to underwater scenarios with high requirements for sensitivity and accuracy [14]. The capacitive MEMS accelerometer uses variable capacitors as sensing components and has the advantages of high sensitivity, simple structure, low noise, and low temperature sensitivity, whereas it is susceptible to parasitic capacitance interference and requires complex detection circuits. Additionally, the non-linear effect of the capacitive accelerometer structure is significant at large displacement, limiting its linear dynamic testing range [15]. Unlike piezoresistive and capacitive sensors, the piezoelectric MEMS accelerometer is a passive device that requires no external power supply. It exhibits excellent piezoelectric coupling, high quality factor, wider linear amplitude range, and lower power consumption, making it widely used in marine resource exploration and inertial sensing systems [16]. Piezoelectric MEMS accelerometers commonly use lead zirconate titanate (PZT), zinc oxide (ZnO), and aluminum nitride (AlN) as thin film materials for electromechanical conversion. As three kinds of typical piezoelectric materials, the piezoelectric coefficients *d*_31_ of AlN and ZnO thin film is 3.4~6.4 pC/N and 5.9~12.4 pC/N, respectively [17]. However, the piezoelectric coefficients *d*_31_ of PZT thin film with high energy density and output voltage is up to 60~223 pC/N [18], tens of times higher than the value of AlN and ZnO. PZT has gained significant interest due to its high dielectric constant, low cost of fabrication, good piezoelectric properties, and convenience to be incorporated into MEMS accelerometers. Several studies have been carried out on piezoelectric MEMS accelerometers. Between 2006 and 2010, Hinichsen et al. [19,20,21] developed a series of PZT piezoelectric accelerometers based on beam structure and the inertial mass unit. Firstly, they fabricated and characterized a micromachined accelerometer with four beams and four inertial unit structures. Subsequently, they presented a theoretical model of a PZT MEMS accelerometer based on four-cantilever beams and single inertial unit. In 2019, Xu et al. [22] fabricated a PZT four-cantilever beams accelerometer with interdigital electrodes structure. The results indicated that a higher voltage output can be achieved with a smaller filling and electrode interspace. Recently, in 2021, Lee et al. [23] proposed and fabricated a piezoelectric MEMS accelerometer on Si substrates using photolithography and sol-gel PNZT. The accelerometer exhibited a sensitivity of 16.8 mV/g at 200 Hz. According to the above research, the four-cantilever beam structure has advantages, such as high sensitivity, wide frequency range, improved linearity, low power consumption, and easy fabrication, making it a popular choice for piezoelectric accelerometers used in various applications [8].

Research has focused on the theoretical analysis and functional verification of piezoelectric MEMS accelerometers in vector hydrophones to improve their design and performance. In 2017, Kim et al. [24] developed a PMN-PT crystal vibrator for vector hydrophones to measure the magnitude and direction of acoustic signals. The voltage response equation of the PMN-PT crystal vibrator was derived to guide the design of shear moding accelerometers in vector hydrophones. However, the accelerometer was not packaged as a hydrophone for testing and verification. In 2018, Seonghun et al. [25] proposed a shear-type accelerometer for a vector hydrophone, which was applied to a towed array sonar system. The minimum receiving voltage sensitivity (RVS) of the hydrophone demonstrated a remarkable capability of up to −201.4 dB at the operating frequency range, achieving the highest RVS over the frequency range. It should be noted that a single hydrophone can only test directivity in one direction. In 2020, Cho et al. [26] developed a PIN-PMN-PT piezoelectric accelerometer for vector hydrophone. Results showed the sensitivity of the accelerometer and hydrophone to be −199 dB and −196 dB, respectively. The sensor produced the expected cardioid directivity pattern across the operating frequency range. Although the basic functionality of the proposed vector sensor was confirmed, further research should be conducted to enhance its sensitivity. In 2022, Roh et al. [27] designed and optimized a shear-type accelerometer to improve the performance of vector hydrophones. The RVS of the hydrophone is −204.9 dB. Coupled with an omnidirectional hydrophone, the dipole response can generate a cardioid directivity pattern that detects both the magnitude and direction of external sound sources. Although significant studies have been made in the design and development of vector hydrophones, there are still challenges that need to be addressed. For instance, more accelerometer research is required to increase the sensitivity of vector hydrophones, particularly at a low-frequency range. In addition, the translation of theoretical designs into viable sensors is still a hurdle that needs to be overcome.

Therefore, based on a cross-beam integrated inertial mass unit structure, this paper developed a piezoelectric MEMS accelerometer (PMA) for vector hydrophones. Firstly, the impact of the geometric dimensions of the cantilever beam on resonance frequency and stress distribution was numerically analyzed, and its parameters were optimized. In addition, the overall structure of the piezoelectric microstructure was designed, and the manufacturing process of the device was proposed. PZT piezoelectric films were prepared using sol-gel technology as the sensitive unit, and MEMS technology was used to manufacture the acceleration microdevice. The MEMS accelerometer was packaged on a vibration test platform in order to investigate and analyze the performance of the PMA within a range of input acceleration and frequency. Finally, the accelerometer was encapsulated as a piezoelectric vector hydrophone (PVH) to investigate the sensitivity and directivity in a calibration system. The PVH with PMA has high sensitivity and good directivity.

## 2. Theoretical

**Figure 1:**
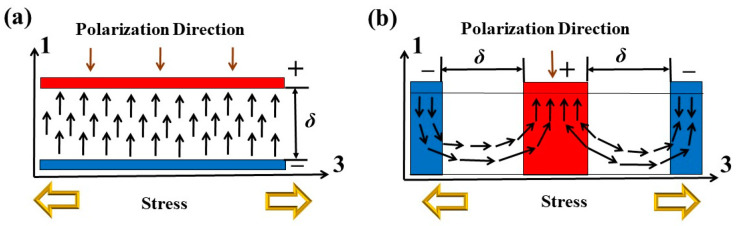
Electromechanical conversion mode of piezoelectric material: (**a**) longitudinal mode *d*_31_ and (**b**) transverse mode *d*_33_.

Piezoelectric materials play an important role in the sensor dynamic response. Under different external forces, piezoelectric materials exhibit different types of electromechanical conversion modes, as shown in Figure 1. Most piezoelectric materials used in energy conversion sensors have a clear polar axis, and the different direction between external forces and the polar axis affects the material performance [28]. According to the working mode, the piezoelectric system mainly includes *d*_31_ working mode and *d*_33_ working mode. In the *d*_31_ mode, the external forces applied to the piezoelectric material are perpendicular to the electric field polarization direction. In other words, direction 1 is the direction of generating the coupled electric field, and direction 3 is the direction of applying the force, forming a sandwich structure with top and bottom electrodes. In the *d*_33_ mode, the piezoelectric material is subjected to force, and the polarized electric field is along the direction 3, forming a plane–bimorph structure. In the *d*_31_ and *d*_33_ working modes, the charge in the piezoelectric layer is induced to be perpendicular and parallel to the strain direction, respectively. The open-circuit voltage *V_oc_* and transferred charge *Q_oc_* of the piezoelectric layer can be expressed as follows [29,30]:

(1) $$\left\{\begin{array}{l} V_{oc}=\frac{\sigma_{3i}d_{3i}t}{\varepsilon_{r}\varepsilon_{0}}\\ Q_{oc}=-\sigma_{3i}d_{3i}A_{3i} \end{array}\right.$$

where *σ*_3*i*_ (*i* = 1, 3) represents stress; *t* represents the piezoelectric layer thickness; *d*_3*i*_ (*i* = 1, 3) represents the piezoelectric coefficient; *ε_r_* and *ε*_0_ represent the relative dielectric constant and vacuum permittivity, respectively; and *A*_3*i*_ (*i* = 1, 3) represents the effective area of the electrodes.

In Equation (1), *V_oc_* is proportional to *σ*_3*i*_, *d*_3*i*_, and *t*, and *Q_oc_* is proportional to *σ*_3*i*_. Obviously, the piezoelectric material performance depends on the type of working mode. Normally, the piezoelectric film is usually very thin to reduce the geometric dimensions of the sensor structure. The thickness of the piezoelectric film in *d*_31_ is shorter than *d*_33_. The *d*_31_ working mode has the advantage of higher voltage output, while *d*_33_ has a larger current output. Meanwhile, the *d*_31_ PZT film will produce larger mechanical strain during vibration.

**Figure 2:**
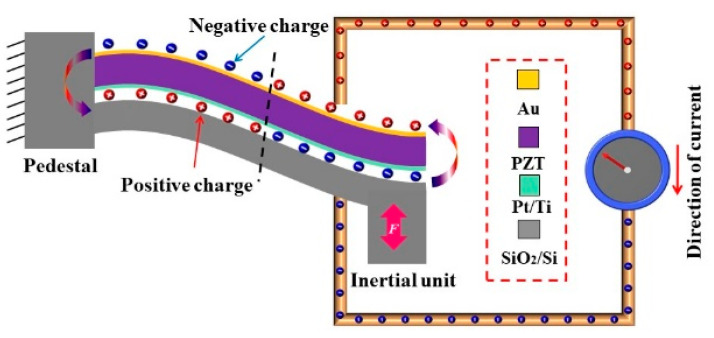
The electrical charges generated on the single cantilever beam.

Due to the strain generated inside the cantilever beam, which is a relatively simple structure during vibration, the piezoelectric cantilever beam is the key structure for collecting mechanical energy from vibration. According to the piezoelectric working mode and mechanical vibration, an external force can drive the cantilever beam to vibrate. Vibration causes polarization changes in the piezoelectric film on the cantilever beam, resulting in voltage signals on the surface of the thin film. The compressive stress on the upper surface and the tensile stress on the lower surface generate an electric charge of opposite polarity on both surfaces of the film, causing opposite deformation of the two parts of a single cantilever beam. The stress difference on the two surfaces of the cantilever beam leads to the distribution of charges between the two surfaces, as shown in Figure 2. In this structure, there are two piezoelectric units connected in series, one lower electrode and two disconnected upper electrodes [31].

## 3. Design and Simulation

### 3.1. Design of the Sensor Microstructure

**Figure 3:**
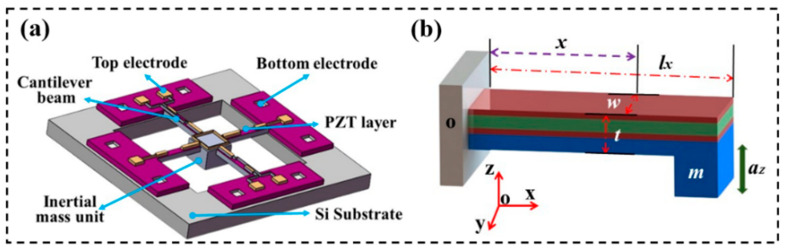
Microstructure model: (**a**) the microstructure with four cantilever beams and an inertial mass unit, (**b**) coordinates and parameters of a single cantilever beam.

According to the above theory, we designed a MEMS accelerometer with four cantilever beams and a central inertial unit based on Au/PZT/Pt/Si, as shown in Figure 3a. The vibration energy is converted into electricity by the *d*_31_ PZT film. When the central inertial unit is subjected to vibration, the cantilever beam experiences stress in the opposite direction, generating an electrical signal with opposite phase. This multichannel output improves the stability of the signal from the device. The upper and lower electrodes are made of low-resistivity Au and Pt, respectively. Due to the constraints of the four-beam structure, when the transverse swing of the mass unit is ignored, acceleration triggers the vibration of the inertial mass unit. This vibration drives the cantilever beam to swing, resulting in the generation of strain in the PZT film integrated on the suspended beam, which then produces an electrical signal output reflecting the value of the acceleration. Similar to other forms of accelerators, mechanical properties are determined by the structural parameters of the beam. To establish a Cartesian coordinate system (o-x-y-z), shown in Figure 3b, the length, width, and thickness of the beam are denoted by *l*, *w*, and *t*, respectively. The central coordinate of the inertial unit is located at *x* = *l*. In the theoretical model, the bending of the beam mass unit and inertial mass unit is ignored, and the effect of mass unit motion without rotation is guaranteed. Without considering the gravitational acceleration, the mass unit m will move downward or upward under an external acceleration *a_z_* in the z-axis direction. The four cantilever beams undergo the same deformation due to the symmetry of the microstructure. In accordance with small deflection theory, the torque balance equation on the beam is expressed as [32]:

(2) $$EI_{x}\omega^{\prime \prime }\left(x\right)=F\left(l_{x}-x\right)-M_{0}$$

where *x* represents the distance from the constraint end to any point on the beam. E is Young’s modulus of Si, *I_x_* is the moment of inertia along the x-axis, *ω*(*x*) is the displacement of *l_x_* along the z-axis, *F* is the reaction force, and *M*_0_ is restriction moment.

(3) $$I_{x}=wt^{3}/12$$

The concentrated force *F* on the cantilever beam can be determined as:

(4) $$F=ma_{Z}/4$$

where *m* represents the mass of the inertia unit.

(5) $$\omega \left(x\right)=\int \int_{0}^{l_{x}}\left(\frac{3ma_{Z}}{EI}dx\right)dx+Ax+B$$

*A* and *B* are constants. The boundary condition is expressed as:

(6) $$\omega \left(0\right)=\omega^{\prime }\left(0\right)=0$$

From Equations (3), (5), and (6), the displacement of *l_x_* along the z-axis direction is expressed as:

(7) $$\omega \left(x\right)=\frac{12ma_{Z}}{4Ewt^{3}}\left(\frac{1}{2}l_{x}x^{2}-\frac{1}{6}x^{3}\right)$$

When *x* = *l_x_*, the displacement can be expressed as:

(8) $$\omega \left(l_{x}\right)=\frac{ma_{Z}l_{x}{}^{3}}{Ewt^{3}}$$

In the *x*-axis direction, the maximum stress of the cantilever beam can be expressed as:

(9) $$\sigma_{\max}=\frac{Fd}{I_{x}}=\frac{3l_{x}}{2wt^{2}}ma_{Z}$$

where *d* = *h*/2 is the distance between the neutral plane of the cantilever beam and the piezoelectric layer. According to Hooke’s law, the displacement of the inertial unit is equal to the displacement of the cantilever. The elastic constant can be expressed as:

(10) $${\left(\frac{k}{m}\right)}^{2}=\frac{ma_{Z}}{\omega \left(x\right)}=\int_{0}^{l_{x}}EI{\left[\omega^{\prime \prime }\left(x\right)\right]}^{2}dx/\frac{1}{2}m{\left[\omega \left(l_{x}\right)\right]}^{2}$$

(11) $$\frac{k}{m}=\sqrt{2Ewt^{3}/4ml_{x}^{3}}$$

where *k* represents the elastic coefficient of the cantilever beam. The resonance frequency of the cantilever beam can be expressed as:

(12) $$f=\frac{1}{2\pi }\sqrt{\frac{k}{m}}=\frac{1}{2\pi }\sqrt{Ewh^{3}/4ml_{x}^{3}}$$

Positive and negative charges appear on the top and bottom surfaces of the PZT film, and the charge quantity *q* is expressed as:

(13) $$q=d_{31}\sigma_{\max}$$

where the *d*_31_ is the piezoelectric coefficient of normal stress, *σ*_max_ is the normal stress. In light of the definition, the capacitance *C* between the top and bottom electrode can be obtained as follows:

(14) C=q/V

By substituting Equations (9) and (13) into Equation (14), the output voltage *V* is expressed as:

(15) $$V=d_{31}\frac{3l_{x}}{2wt^{2}C}ma_{Z}$$

Combined with Equations (14) and (15), the sensitivity *S* of the PZT element can be expressed as:

(16) $$S=\frac{\Delta V}{\Delta a}=d_{31}\frac{3l_{x}m}{2wt^{2}C}$$

According to the above analysis, to improve the output voltage sensitivity, it is recommended to increase the inertial unit mass and beam length, while decreasing the beam thickness and width. During the size determination of the MEMS accelerometer, factors include the microdevice sensitivity and microstructure working frequency bandwidth. Microdevice sensitivity is proportional to the cantilever beam stress, with higher stress leading to greater sensitivity and vice versa. Broaden the microstructure working frequency bandwidth by increasing the natural frequency of the sensitive microdevice, but fixed structure size means the sensitivity and frequency are contradictory. PVH performance bandwidth is mainly determined by the natural frequency of the sensing structure, and increased natural frequency leads to the wider bandwidth of PVH. Hence, it is necessary to conduct further static stress to determine the sensor dimensions.

### 3.2. Simulation

**Figure 4:**
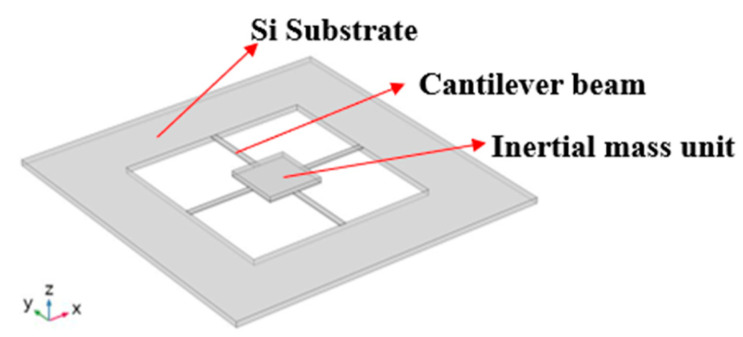
Microstructural model in COMSOL Multiphysics.

To consider the performance of the microstructure before fabrication, Finite Element Analysis was performed using COMSOL Multiphysics 6.1 software, as shown in Figure 4. In the design of beam and inertial mass unit structure, not only the material settings, but also the physical settings of the Multiphysics simulation interface of the piezoelectric components are used; the simulation parameters of the principal material are listed in Table 1.

**Figure 5:**
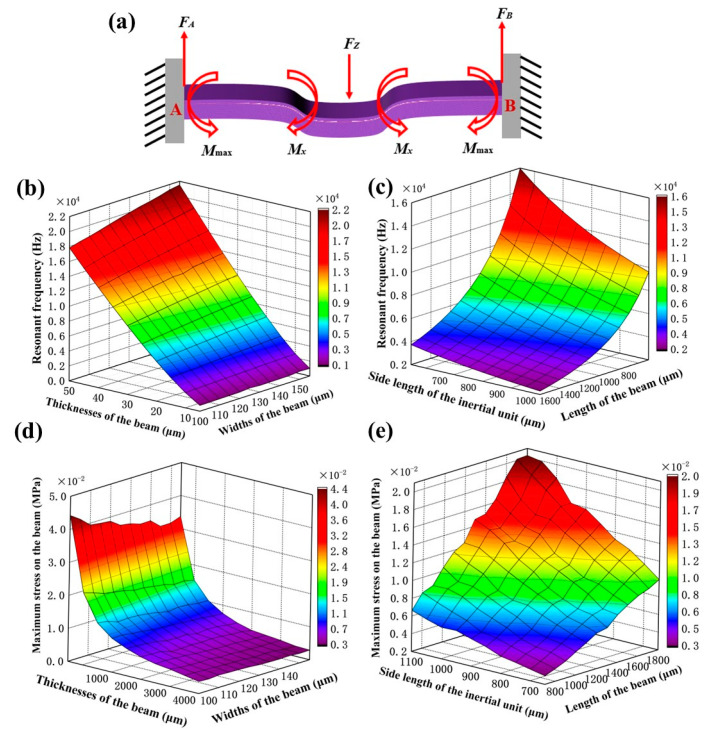
The effects of microstructural parameters on the resonant frequency and maximum stress: (**a**) the force analysis and deflection diagram of the microstructure, (**b**) relationship of the resonant frequency on the width and thickness of the beam, (**c**) relationship of the resonant frequency on the beam length and the inertial mass unit, (**d**) relationship of the maximum stress on the width and thickness of the beam, (**e**) relationship of the maximum stress on the beam length and the inertial mass unit.

Design variables are the length of cantilever beams, widths of cantilever beams, thicknesses of cantilever beams, and the side length of the inertial mass unit. Under the action of the external force *F_z_*, the relationship of the microstructure dimensions, the resonant frequency, and the stress is shown in Figure 5. Figure 5a shows the force analysis and deflection diagram of the microstructure. By varying the microstructure dimensions, which include the length, width, thickness of the cantilever beams, and the side length of the inertial mass unit, the resonant frequency and the maximum stress can be changed. Moreover, the effect of microstructure dimensions is being analyzed using COMSOL Multiphysics 6.1 through parametric scanning methods. Considering the restrictions of the manufacturing process and the sensor microstructure parameters, Table 2 shows the parameter setting for the scanning analysis. The other components are set as free moving parts, while the base is set as a fixed constraint. Figure 5b,c show that the increase of the width and thickness of the cantilever beam will increase the resonant frequency. On the contrary, the increase of the length of the cantilever beam or the inertial mass unit will keep the resonant frequency decreasing continuously. Using a parametric scanning simulation with the above parameters, the resonant frequency of approximately 4 kHz can be obtained. For an input acceleration of 1 g and a frequency of 1 kHz, the effects of the dimensions on the maximum stresses are shown in Figure 5d,e. The maximum stress decreases as the width and thickness of the cantilever beam increase, and increases as the inertial mass unit or side length of the beam increases. Although the maximum stress fluctuates, the overall trends of performance changes are consistent and well below the fatigue strength of the silicon substrate. From the analysis of the natural frequency and maximum stress, the microstructure dimensions can be determined as shown in Table 3.

**Figure 6:**
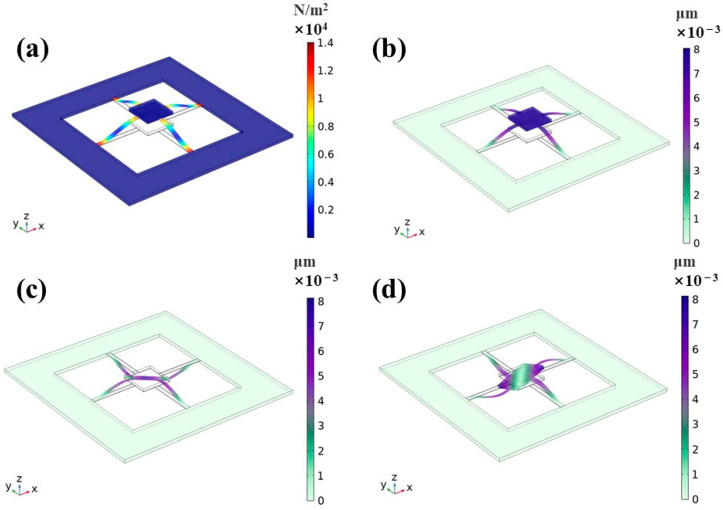
Modal analysis using COMSOL Multiphysics: (**a**) the deformation and stress distribution of the microstructure, (**b**) the 1st-ordered resonance, (**c**) the 2nd-ordered resonance, and (**d**) the 3rd-ordered resonance.

When the geometric dimensions are obtained, the stress distribution and modal analyses can be performed in Figure 6. The acceleration of 1 g was loaded along the z-axis direction to the inertial mass unit; the stress distribution and deformation of the microstructure are showed in Figure 6a. It can be seen that the region of stress concentration is mainly distributed at both ends of the cantilever beam. Moreover, the stress near the support frame areas is more concentrated, which is in line with the expected design. Therefore, the piezoelectric unit should be arranged in the stress concentration area as much as possible to produce a larger output voltage. Figure 6b–d show that the 1st-, 2nd-, and 3rd-order resonance frequencies are 2298 Hz, 12,198 Hz, and 12,199 Hz, respectively. Whereas the 1st resonance mode exhibits vertical displacement, the 2nd and 3rd resonance modes exhibit torsional displacement in different directions. The working frequency of the accelerometer should be lower than the 1st-order resonance frequency to ensure stability of performance.

## 4. Fabrication and Testing

### 4.1. Fabrication of the MEMS Acceleration Microstructure

**Figure 7:**
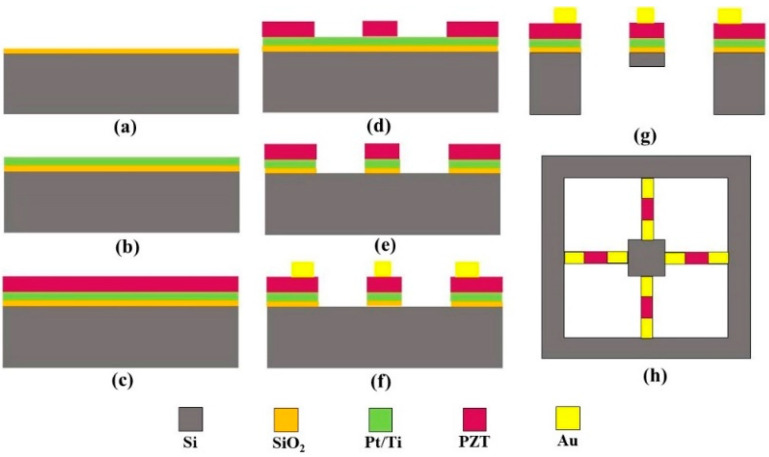
Fabrication scheme of the MEMS piezoelectric microstructure: (**a**) SiO_2_ grown by thermal oxidation. (**b**) Pt/Ti sputtered onto the SiO_2_/Si substrate. (**c**) PZT grown by sol-gel combined with the annealing process. (**d**,**e**) PZT, Pt/Ti, and SiO_2_ pattern produced by photolithography and IBE. (**f**) Au/Ti was sputtered, followed by a peel-off process. (**g**,**h**) Cantilever beams and mass fabrication using lithography and etching by the RIE process.

The acceleration microstructure was fabricated in a clean laboratory. The main processes include thermal oxidation, sputtering, sol-gel, photolithography, ion beam etching (IBE), and reactive ion etching (RIE). The fabrication scheme of the MEMS piezoelectric microstructure is shown in Figure 7.

I. Thermal oxidation was used to grow 330 nm SiO_2_ on the silicon wafer surface as a transition layer (Figure 7a).

II. Pt/Ti was sputtered onto the SiO_2_/Si substrate as the bottom electrode (Figure 7b).

III. A 1 μm thick PZT (PbZr_0.53_Ti_0.47_O_3_) piezoelectric film was grown on the Pt/Ti/SiO_2_/Si (100) substrate using sol-gel combined with the annealing process as the functional layer. The annealing process is completed in a tubular annealing furnace in an ultra-clean room. The annealing process is divided into three sections: the first section (low-temperature section) is 450 °C for 10 min; the second section (high-temperature section) is 650 °C for 10 min; and in the third section, 10% excess lead (Pb) is added at 700 °C for 30 min (supplement Pb lost in the annealing process). After annealing, a layer of PZT film with preferential crystallization growth is obtained, and more than 10 steps are conducted continuously to form 10 layers of PZT film (Figure 7c).

IV. The IBE process was used to sequentially etch PZT, Pt/Ti, and SiO_2_ to pattern the PZT piezoelectric unit, Pt/Ti bottom electrode, and SiO_2_ layer (Figure 7d,e).

V. Au/Ti was sputtered, followed by a peel-off process to complete the preparation of the top metal electrode on the PZT surface (Figure 7f).

VI. The RIE process was used to etch the front and back of the silicon to determine the thickness of the cantilever and release the four cantilevers (Figure 7g).

The acceleration microstructure manufactured through the above process is shown in Figure 7h. In the fabrication process of the microstructure, mature UV photolithography and dry etching techniques were used to ensure the accuracy of the structural dimensions of the cantilevers and the inertial mass unit as much as possible, which improved the stability of the sensor fabrication process and the yield of the device, laying a device foundation for the integration of hydrophones and the improvement of the sensor performance.

**Figure 8:**
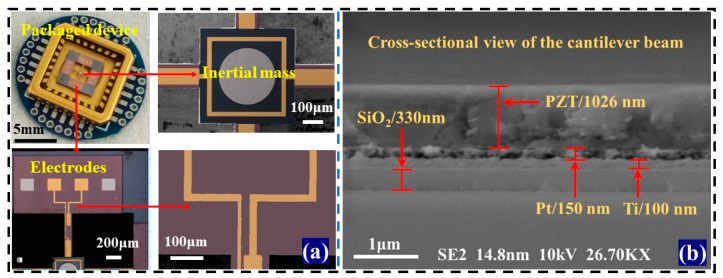
Morphological characterization of the PMA: (**a**) photos of the packaging equipment, indicating the electrodes on the base and cantilever beam, respectively, and (**b**) SEM images of cantilever beam cross-section, representing different thicknesses of PZT, Pt/Ti, SiO_2_, and silicon wafer layers, respectively.

After the fabrication of PMA, scanning electron microscopy (FE-SEM, SUPPA-55, Germany, SE2, 14.8 nm, 10 kV, 26.79 KX) was used to obtain the accelerometer morphology in an ultra-clean room and room temperature, as shown in Figure 8. The microstructure has a clear and smooth boundary, and the surface is clean without stains. The cantilever beam has a width of 110.82 μm, which is consistent with the design dimensions. In addition, the small modulus of the cantilever beam is beneficial for the cantilever beam to bend under external vibration or impact force, thereby generating charges on different surfaces. The MEMS sensors ensure their long-term stability due to their sturdy mechanical structure. The microstructure is encapsulated on the tube shell at room temperature, and then pasted onto a printed circuit board (PCB) to form a device/tube shell/PCB packaging structure. The upper and lower electrodes are wire bonded separately to complete the packaging of the sensor. As shown in Figure 8a, the boundaries of the four cantilever beams are clear, and the structure is symmetrical. The metal electrode pattern is intact, and the inertial mass unit is in a suspended state, effectively avoiding interference between the mass unit and the bottom surface of the sensor during vibration. As can be seen from Figure 8b, the PZT film deposited on the cantilever beam has a thickness of about 1 μm, and the interface between each layer of the material is clearly defined. The PZT film is well bonded and adhered to the Pt/Ti/SiO_2_/Si substrate, resulting in a good and clear interface between the PZT film and the substrate. In addition, the cantilever beam has a small modulus, which helps the beam to bend under external vibrations or impacts, thereby generating charges on different surfaces. PMA ensures long-term stability due to its sturdy mechanical structure.

### 4.2. Performance of the MEMS Accelerometer

**Figure 9:**
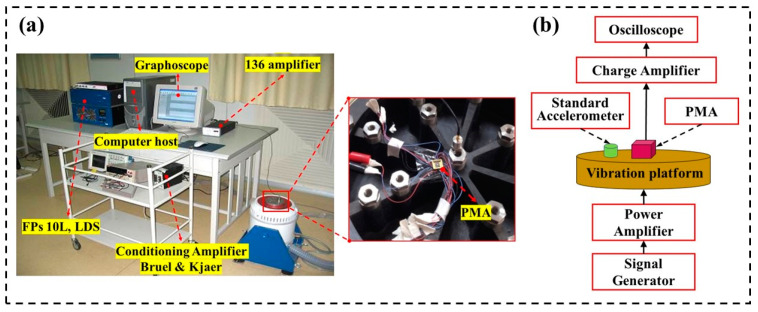
The vibration measurement system of the PMA: (**a**) vibration measuring platform, (**b**) flow chart of measurement system.

The vibration measurement system of the PMA is shown in Figure 9. The measuring platform is shown in Figure 9a. The vibration calibration system developed by B&K company is used to provide vibration interference of different frequencies for PMA. A function generator (Agilent 33522 A, Agilent Technologies, Santa Clara, CA, USA) generates a sine wave signal, which is amplified by a power 136 amplifier, and finally fed into the vibration exciter to generate the vibrations in the required directions [33]. The flow chart of measurement system is shown in Figure 9b. Both the packaged PMA and the standard accelerometer are fixed on the vibration platform to ensure that the maximum output direction is perpendicular to the platform surface. The test system generates a sine wave signal through the signal generator, which is enhanced by the power amplifier. The amplified signal drives the shaker to vibrate regularly, and the vibration frequency is consistent with the sine signal. After passing through the charge amplifier, the output voltage of the PMA is input to an oscilloscope to display the output voltage. Additionally, the power amplifier is set so that the vibration platform produces a certain amount of acceleration, as measured by the standard accelerometer. The fixed acceleration can be applied to the PMA to observe its output signal.

**Figure 10:**
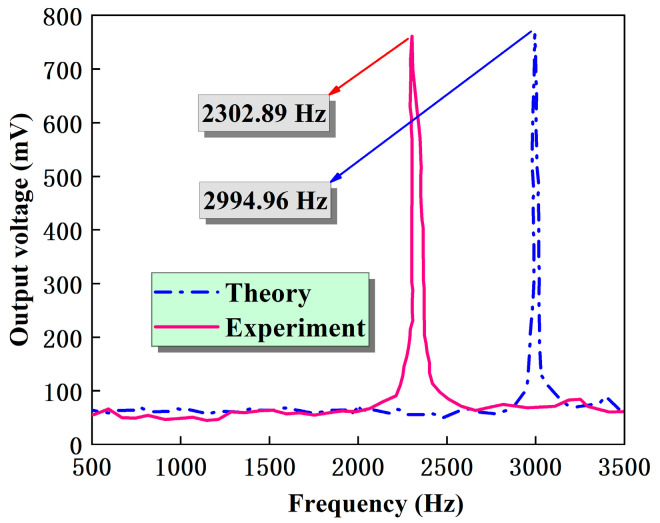
Resonant frequency curve of PMA.

The resonance frequency of the PMA is shown in Figure 10. The output of the PMA increases with increasing frequency and reaches its maximum value at about 2300 Hz, indicating that this frequency is the resonant point of the PMA. After passing through the resonant point, the output voltage drops sharply. Nevertheless, there is still a certain difference between the measured and designed target frequency of about 3000 Hz. This difference may be attributed to the MEMS fabrication process, where there is a certain difference between the actual geometric dimensions and the designed ones of the cantilever beams and the central inertial unit. It is impossible to achieve a rigid installation between the sensor and vibration platform. In addition, the damping coefficient around the encapsulated acceleration sensor in air may also cause the frequency difference mentioned above [34]. In any case, the resonant frequency results prove the effectiveness of our designed accelerometer.

**Figure 11:**
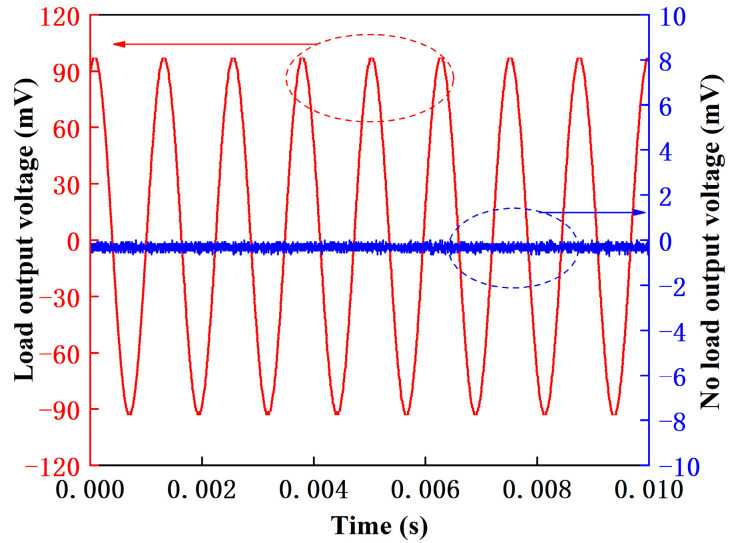
No-load and load output voltage curve.

A single cantilever beam was analyzed to study the noise impact of PMA that was measured on a vibration testing system. The accelerometer was installed on the excitation platform to test the sensor frequency response under load. On the other hand, the sensor was tested without being installed on the excitation table to measure its frequency response under no load. Under a 1000 Hz vibration frequency input, the results of the noise signal and output electrical signal comparison test are shown in Figure 11. The blue curve is for the no-load condition, and the peak-to-peak voltage value of the testing system is about 86 μV. The red curve is for the load condition, and the peak-to-peak voltage value of the piezoelectric element is about 183.4 mV, which is much larger than the output voltage value under no load, differing by about three orders of magnitude. The PMA is a passive piezoelectric sensor, and noise mainly comes from the testing system. The noise is relatively small compared to the output signal, so it can be ignored. The noise has little impact on the output signal of the piezoelectric element, demonstrating good anti-interference ability and reducing the influence of the noise gradient in the environment on the sensor detection performance.

**Figure 12:**
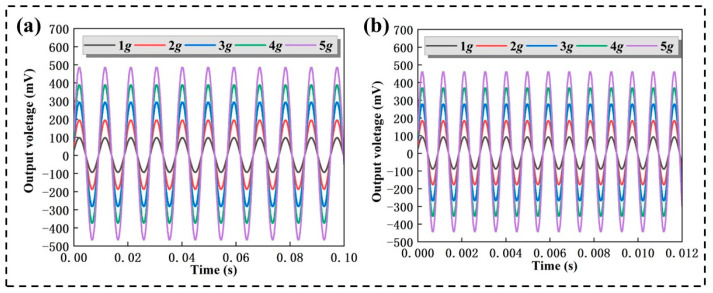
Output voltage curves of the PMA under different accelerations from 1 g to 5 g at (**a**) 100 Hz and (**b**) 1000 Hz.

In addition, the influence of different accelerations and frequencies on the acceleration performance is discussed. The voltage curve of the PMA is shown in Figure 12. By adjusting the power amplitude, the vibration platform was increased from 1 g to 5 g in steps of 1 g, and excited by a sine wave signal with input frequency at 100 Hz and 1000 Hz. Figure 12a,b are shown as output voltage curves of the PMA at 100 Hz and 1000 Hz, respectively. The results show that as the acceleration increases, the output signal is increased proportionally. The perfect response indicates the high-performance sensitivity of the PMA, which is able to meet the demand of hydrophones.

**Figure 13:**
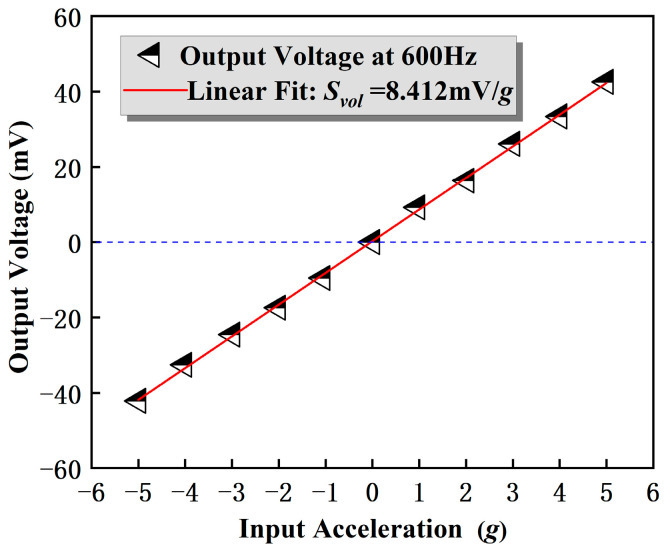
Output voltage sensitivity of the PMA at 600 Hz, ranging from −5 g to 5 g.

Finally, sensitivity testing was performed on the accelerometer. The vibration platform was set to start from 0 g and gradually increase to 5 g in steps. Each acceleration value was loaded with a negative acceleration measurement for 2 min in the same way, i.e., applying −5~5 g acceleration to the sensor under testing. The output voltage results at each acceleration were exported from the data interface of the digital oscilloscope. Then, the data were linearly fitted using the least squares method, and Figure 13 shows the output curve of the test, which shows the relationship between the output voltage and PMA values. The relationship between the output voltage of a single cantilever beam and the acceleration value is plotted at an excitation frequency of 600 Hz. The results showed that the input acceleration and output voltage of the PMA had a linear relationship, and the function expression was *V* = *ka* + *V_o_* = 8.412*a* − 0.212 (where *k* is the slope of the line, and *V_o_* is the voltage intercept). The linear correlation coefficient R^2^ of the sensor’s single cantilever beam was 0.9997, approaching 1. Therefore, the sensor has good acceleration response linearity and passive stability. The slope is equal to its prime sensitivity, which measures the output voltage per unit of gravitational force applied. In this case, the slope is found to be 8.412 mV/g for a single beam. Hence, since all four beams on the accelerometer are coupled in parallel, the sensitivity of the entire sensor is 33.65 mV/g.

### 4.3. Performance of the MEMS Hydrophone

**Figure 14:**
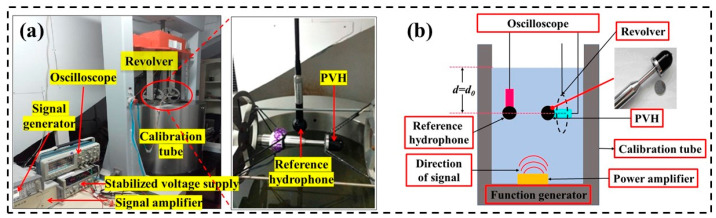
Measuring calibration system of the PVH: (**a**) test calibration system, (**b**) schematic diagram of hydrophone calibration device.

To investigate the performance of the PVH for vector hydrophone, which was encapsulated inside the polyurethane waterproof cap of the vector hydrophone [35], the standard wave tube calibration system was used to measure the sensitivity and directionality of the vector hydrophone [36], as shown in Figure 14. The standing wave tube measurement site is shown in Figure 14a. The PVH and a standard hydrophone were placed in a free sound field simultaneously. The output signals of the two sensors were compared with the standard hydrophone used as a reference to obtain the received sensitivity of the PVH. The sensitivity of the PVH was calibrated to the standard hydrophone (where sensitivity of the standard hydrophone is −180 dB (0 dB = 1 V/μPa)). The schematic diagram of the testing system is shown in Figure 14b. The PVH was installed on a mechanical rotating device, which had four orthogonal fixed ends for securing it. A sine wave with a peak-to-peak voltage of 1 V and 20 dB amplification was generated as the signal source, and the frequency range was swept from 1/3 octave to 2 kHz, with a sampling frequency of 20~2000 Hz. The elevation control console was adjusted to place the standard hydrophone and the uncalibrated hydrophone at distances *d*_0_ and d from the water surface, respectively. The open-circuit out voltage *e*_0_ of the standard hydrophone was measured to obtain its sound pressure *p*_0_. Similarly, the open-circuit output voltage *e_x_* of the uncalibrated hydrophone was measured, and the sensitivity of PVH at each frequency point was calculated based on the sound pressure information obtained at the location of the standard hydrophone and the two sets of measured values. According to the above analysis of the testing process, the values need to be recorded in the sensitivity calibration include: the open-circuit output voltage of PVH (*e_x_*), the open-circuit output voltage of the standard hydrophone (*e*_0_), and the calibration value of the standard hydrophone sensitivity in the free field (*M*_0_). According to the definition of the hydrophone voltage sensitivity in the free field, *M*_0_ is expressed as [4]:

(17) $$M_{0}=e_{0}/p_{0}$$

The sensitivity of PVH is expressed as:

(18) $$M_{x}={e_{x}/p}_{x}$$

where *p*_0_ and *p_x_* are the sound pressures at the acoustic centers of the standard hydrophone and PVH, respectively, before placing them in the sound field. *d* and *d*_0_ are the depths of the centers of the PVH and the standard hydrophone mounted on the mechanical rotation device in the water, respectively. The sound pressure at any point in the standing wave barrel satisfies the relationship *p* ∝ sin *kd*. In the testing process of this paper, the PVH and standard hydrophone are placed on the same horizontal plane (*d = d*_0_). Therefore, the sensitivity of the PVH is given by:

(19) $$M_{x}=20\mathrm{lg}(\frac{e_{x}}{e_{0}}\frac{\sin kd}{\cos kd_{0}})+M_{0}$$

**Figure 15:**
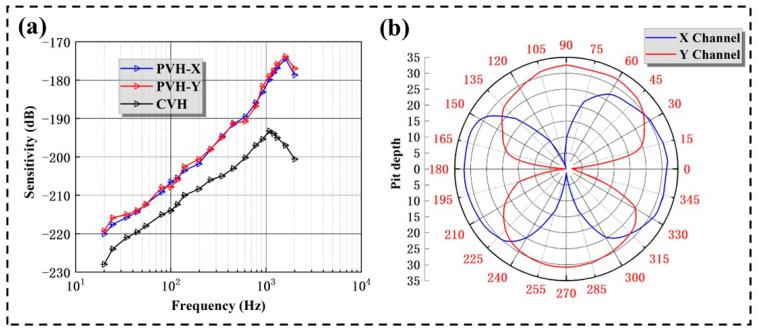
Measuring calibration system and performance of the PVH: (**a**) comparison chart of hydrophone sensitivity curve and (**b**) the “8” character directivity diagram at 800 Hz.

According to Equation (19), the sensitivity of the PVH can be obtained, and the sensitivity curve can be plotted based on the data. Figure 15a shows the x-axis and y-axis frequency response curve of the PVH. It can be seen that the PVH has good low-frequency characteristics, with a sensitivity of −178.99 dB@1000 Hz (0 dB = 1 V/μPa) and a frequency bandwidth of 20~1100 Hz, 11 dB higher than the bionic cilia MEMS vector hydrophone (CVH) at the same frequency [7], suitable for quiet underwater applications.

Theoretically, the various channels of a vector hydrophone should have cosine directivity independent of frequency. The PVH is suspended in a rotating frame within a standing wave tube, with the coordinate axis of the channel to be tested parallel to the axis of the standing wave tube and pointing towards the transmitting transducer (as is shown in Figure 14a). While keeping the output power and frequency of the transmitting transducer stable, the PVH was rotated around a circle using the rotation device, and the output voltage values corresponding to different angles were recorded. Finally, the results are normalized and expressed in logarithmic form to obtain the directivity curve of the PVH at this frequency. The rotational speed of the rotary device is 120°/min, and the recording interval of the recorder is 0.2 s, which means that the angle interval for the directivity test is 0.4°. As can be seen from Figure 15b, the results show that the PVH has a good and smooth “8”-shape cosine directivity at 800 Hz. The depths of the sensitivity notch for the X and Y channels are 32.46 dB and 32.08 dB, respectively. The depth of the sensitivity notch for each channel is greater than 32 dB, which meets the application requirements.

## 5. Conclusions

In this study, a piezoelectric MEMS accelerometer was designed and fabricated for vector hydrophones with four-cantilever beam integrated inertial mass unit structure manufactured using MEMS technology. The cantilever beam of PMA is comprised of Si, SiO_2_, Pt/Ti, PZT, and Au, arranged from bottom to top. The high dielectric constant PZT film was employed to measure the transverse sensitivity of the *d*_31_ working mode. The accelerometer was tested on a vibration platform. The results indicate that the resonant frequency of the PMA is 2300 Hz, with high anti-interference ability, good response linearity, and passive stability. The accelerometer exhibits a perfect response to the input signals, indicating high sensitivity of 33.65 mV/g at 600 Hz. Additionally, the PMA was encapsulated inside the polyurethane waterproof cap of the vector hydrophone. The PVH was tested in a standing wave barrel, revealing excellent low-frequency characteristics, with a receiving sensitivity of −178.99 dB@1000 Hz (0 dB = 1 V/μPa), which is 11 dB higher than the bionic cilia MEMS vector hydrophone (CVH). All channels of PVH exhibited good “8” shape directivity. The fabricated piezoelectric MEMS accelerometer has excellent performance and manufacturing feasibility, indicating its potential for widespread use in various fields requiring high-sensitivity acceleration measurement and underwater target detection applications. The future work will focus on investigating the PVH performance in low-temperature deep sea environments.

## Figures and Tables

**Table 1:** Simulation parameters of principal material.

Material	Parameters	Value
Si	Density	2330 kg·m^−3^
	Young’s modulus	190 Gpa
	Poisson’s ratio	0.26
	Dielectric constant	11.9
PZT	Density	7500 kg·m^−3^
	Young’s modulus	75 Gpa
	Poisson’s ratio	0.32
	Effective Coupling Co-efficient	35 k^2^%
	Curie temperature	365 °C
	Elastic modulus	5 × 10^10^ N/m^2^
	Tensile modulus	2 × 10^7^ N/m^2^
	Dielectric constant	1300
	Piezoelectric charge constant *d*_31_	−270 pC/N
	Mechanical quality factor	32

**Table 2:** Parameters of the scanning.

Parameters	Range	Steps
Length of cantilever beams	700~1800	100 μm
Width of cantilever beams	100~150	5 μm
Thicknesses of cantilever beams	10~50	5 μm
Side length of inertial mass unit	600~1100	50 μm

**Table 3:** Dimension parameters of microstructure (Unit: μm).

Parameters for Scanning	Numerical Value
Length of single beams	1100 μm
Width of beams	120 μm
Thicknesses of beams	25 μm
Side length of the inertial unit	700 μm
Thicknesses of PZT	1 μm
Microstructure size	5000 μm × 5000 μm

## Data Availability

Not applicable.
